# When Learning Gets Expensive: Exploring Placement Poverty in Medical Radiation Science (MRS) Students

**DOI:** 10.1002/jmrs.70085

**Published:** 2026-03-27

**Authors:** Min Ku, Stephen Lacey

**Affiliations:** ^1^ Australian Society of Medical Imaging and Radiation Therapy (ASMIRT) Melbourne Australia

## Abstract

ASMIRT recognises the urgent need to ensure that financial and logistical barriers faced by students are minimised. Priorities include equitable funding and paid placements, strengthened clinical training, and targeted measures to support workforce wellbeing and retention to secure a sustainable and capable medical radiation workforce.

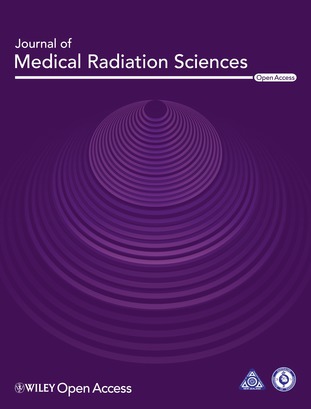

## 1 Introduction

The Australian Society of Medical Imaging and Radiation Therapy (ASMIRT) is the leading professional body representing medical radiation practitioners in Australia. We are dedicated to upholding the highest standards of practice and advancing excellence in medical radiation science. ASMIRT empowers practitioners to deliver outstanding patient care through leadership in advocacy, education, professional standards, and research & innovation. Guided by these four pillars, we foster a dynamic community of expert volunteers who actively engage with and support our members.

ASMIRT recognises the urgent need to ensure that financial and logistical barriers faced by students are minimised. Priorities include equitable funding and paid placements, strengthened clinical training, and targeted measures to support workforce wellbeing and retention to secure a sustainable and capable medical radiation workforce.

Financial stress during Work Integrated Learning (WIL) placements, often called “placement poverty”, is well documented across professions but has received little attention in the Medical Radiation Professions. Braithwaite et al., in this issue of the Journal of Medical Radiation Sciences, highlight systemic financial hardship among medical imaging students. Despite a small, single‐institution sample, the study shows that financial pressures undermine wellbeing, mental and physical health, academic performance, and engagement with clinical placements, contributing to attrition and potentially worsening workforce shortages [1].

Braithwaite et al. compare the clinical placement hours required of medical imaging students with those of radiation therapy and podiatry, noting that medical imaging students complete substantially more placement hours, more than double those required in podiatry. However, such direct comparisons are of limited value for two key reasons. First, the competency and capability requirements of each profession are inherently distinct, making equivalence in placement hours neither expected nor appropriate. Second, this comparison implicitly assumes that the placement hour requirements of other professions are adequate, an assumption that is not necessarily supported by evidence.

To ease student financial hardship, some clinical placement sites offer scholarships or paid placements at their discretion. This bypasses the Australian Fair Work Act, which does not require remuneration for non‐employee students [2]. Although beneficial in the short term, such practices blur the line between student and employee and disproportionately disadvantage students from lower socioeconomic backgrounds, those with caring responsibilities, rural and international students. Research shows that the financial burden of unpaid placements intensifies inequities in access to and success within health professional education [3, 4].

Without clear regulatory frameworks, there is a risk that students are being used to fill workforce gaps, often facing excessive hours that compromise academic obligations [5, 6, 7]. Unpaid interns frequently report exploitation and lower satisfaction compared with paid peers [8]. This evidence underscores the need for sector‐wide policy reform to define student status, protections, remuneration, and workload during placements. ASMIRT warns that using students to offset staffing shortages can result in unsupervised work, compromise safety, and create financial liabilities for clinical partners.

## 2 University

Universities play a key role in ensuring clinical placements meet accreditation, educational, and wellbeing standards. Centralised placement allocation via WIL teams improves efficiency but can limit student engagement with academic supervisors. To address this, universities should implement structured “placement plans” co‐developed with students and staff. This approach ensures compulsory rural or regional placements provide high‐quality learning, while also supporting student welfare and financial sustainability, aligning with accreditation requirements and promoting equitable, effective clinical education.

## 3 The Professional Body—ASMIRT

ASMIRT is deeply concerned about the financial, educational, and workforce pressures facing medical radiation science students, the future of Australia's healthcare workforce. Economic pressures in healthcare workplaces flow directly to students, many of whom face significant financial hardship to attend clinical placements. Some are forced into extreme circumstances, such as sleeping in cars or temporary accommodation, to meet the costs of travel, accommodation, and living expenses.

While the Australian Government recently introduced a strategy to support student clinical placements in several essential services, radiation therapy, diagnostic imaging, and nuclear medicine students were excluded. This omission creates substantial and inequitable barriers, particularly for those undertaking rural, regional, or interstate placements, with some students compelled to abandon their studies because they cannot forgo part‐time employment to complete extended, unpaid placements.

ASMIRT has engaged directly with the Australian Government to address this inequity and advocate for policy reforms that recognise and support the vital contribution of medical radiation science students to Australia's future healthcare workforce. Additional financial support is available through various state government programs and the Victorian Medical Radiation Practitioners Education Trust [9], but gaps remain.

These challenges are compounded by declining enrolment and graduation rates. Evidence from Australian clinical settings shows that radiographers and radiation therapists experienced severe workplace stress and anxiety during the COVID‐19 pandemic, with major disruptions to clinical work patterns and professional wellbeing [10]. COVID‐19‐related interruptions to clinical education have also limited students' hands‐on experience, reducing readiness for practice and raising concerns about the preparedness of new graduates to meet professional standards [11, 12].

Coupled with ongoing burnout, staffing shortages, and difficulties retaining experienced practitioners across allied health, these factors indicate that reduced student throughput not only limits the immediate supply of qualified graduates but also exacerbates systemic workforce instability in a sector still recovering from the impacts of the global health crisis.

## 4 “Earn as You Learn” Model

To address ongoing workforce shortages, ASMIRT is closely examining international models that integrate employment and training, particularly the “earn‐while‐you‐learn” apprenticeship approach employed in the United Kingdom (UK) [13]. In the UK, degree apprenticeships in diagnostic and therapeutic radiography embed students directly within clinical departments as employed apprentices, enabling them to earn a salary while completing both academic and practice‐based competencies required for professional registration with the Health and Care Professions Council [14]. These programmes combine on‐the‐job training with university study and are designed to strengthen workforce pipelines, reduce vacancy rates, and improve retention in high‐need clinical areas [15].

Evidence indicates that apprentices make meaningful contributions to service delivery while gaining integrated clinical experience, thereby enhancing recruitment and retention in imaging and allied health services [16]. The literature further highlights that effective apprenticeship programmes rely on strong employer–education partnerships, structured assessments, and workplace support to ensure students achieve professional competence [17]. ASMIRT notes that a similar apprenticeship model previously operated in Australia before the establishment of the current degree‐based entry route and is monitoring a new trial of this model in nuclear medicine, which may inform the feasibility of reintroducing an integrated employment and training pathway in the Australian context.

The following detail various suggestions to support students as they navigate student placements.

## 5 Financial Assistance

In July 2025, the ASMIRT Board introduced a targeted support initiative for final‐year medical radiation science students experiencing financial hardship. The program provides AUD $500 grants to up to 100 students annually to help offset clinical placement costs. This initiative aims to reduce financial barriers, support successful degree completion, and promote equitable access to clinical education while strengthening the future workforce.

## 6 Advocacy

ASMIRT has actively advocated to the Australian Government for full inclusion of allied health disciplines, including medical radiation science, in the Commonwealth Practical Placement initiative. It has emphasised that exclusion disproportionately disadvantages students in diagnostic imaging, radiation therapy, and nuclear medicine, where extended and rural placements create significant financial and logistical burdens with long‐term workforce consequences.

Complementary government measures such as expanded placement funding and student debt reduction help offset travel, accommodation, and living costs while easing graduates' financial load. Evidence indicates these strategies improve student wellbeing, retention, and willingness to work in high‐need areas, strengthening long‐term workforce sustainability [1, 18].

## 7 Student Clinical Placement

Over two decades, ASMIRT has provided access to the Rural Clinical Placement Scheme, which provides targeted support for students undertaking compulsory placements in rural and regional settings. By offering financial assistance for travel and accommodation, the scheme reduces barriers to participation and ensures that students gain exposure to diverse clinical experiences, enhancing their competence and professional confidence. Rural placements also serve as a critical workforce development strategy, fostering student interest in rural practice and addressing chronic staffing shortages in underserved regions [19].

## 8 Student Placement Pantry

In addition to advocacy and funding initiatives, ASMIRT has explored practical supports to ease placement‐related hardship, including a student placement pantry providing essential food and household items particularly for students in high‐cost or remote locations. Given evidence that financial stress undermines academic performance, clinical engagement, and wellbeing [1], this measure offers a direct, preventative response.

Together, advocacy, government placement funding, debt reduction, rural support, and targeted wellbeing initiatives reflect a coordinated strategy to remove financial barriers, promote equitable clinical education, and strengthen the sustainability of Australia's allied health workforce.

## 9 Clinical Placement Partners

Clinical placement partners are central to preparing medical radiation science students for practice, providing supervised, competency‐based training in real‐world clinical settings. Through mentorship, orientation, and workplace supervision, they ensure students develop the skills required to meet accreditation standards while maintaining safe and effective patient care.

Many partners also help mitigate placement‐related hardship through accommodation support, travel allowances, subsidised parking, meals, or flexible scheduling. However, financial assistance must not compromise the quality or breadth of clinical learning. When high‐quality training is combined with practical support, it promotes equity, strengthens student engagement, and supports the development of a competent, work‐ready workforce.

## 10 Conclusion

To ensure a sustainable and capable future medical radiation workforce, urgent action is required to address financial and logistical barriers for students, particularly those undertaking rural, regional, or interstate placements. This includes equitable funding and paid placement opportunities, enhanced clinical training to recover from COVID‐19–related disruptions, and strategies to support workforce wellbeing and retention. By implementing these measures, policymakers can safeguard graduate readiness, strengthen the pipeline of qualified practitioners, and reduce systemic vulnerabilities within Australia's healthcare sector.

The collaboration between universities, professional organisations such as ASMIRT, and clinical placement partners is essential for delivering high‐quality clinical experiences, fostering professional development, and ensuring the sustainability of a competent and resilient medical radiation workforce.

## AI USE DISLOSURE

ChatGPT was used for the final manuscript draft to assist in the reduction of word count, while maintaining consistency of the writing style. No other use of any form of artificial intelligence (including translation, paraphrasing or editing software or generative AI programs) was used in this research or the production of this manuscript. [Correction added on [29 May 2026], after first online publication: ‘’AI USE DISCLOSURE’’ section has been added in this version.]

## Funding

This research did not receive any specific funding.

## Conflicts of Interest

The authors declare no conflicts of interest.

## Linked Articles

This is a linked article to Braithwaite et al. ‘When Work Integrated Learning Costs Too Much: The Hidden Toll of Clinical Placements.’ To view this article, visit https://doi.org/10.1002/jmrs.70032.

## Data Availability

Data sharing not applicable to this article as no datasets were generated or analysed during the current study.

## References

[1] V. Braithwaite , T. Gunn , P. Rowntree , and J. Singleton , “When Work Integrated Learning Costs Too Much: The Hidden Toll of Clinical Placements,” Journal of Medical Radiation Sciences (2026). 10.1002/jmrs.70032. [Correction added on [29 May 2026], after first online publication: Reference has been updated to reflect the correct article for the editorial] PMC13238703 41216691

[2] Fair Work Ombudsman , “Student placements,” https://www.fairwork.gov.au/sites/default/files/migration/723/Student‐placements.pdf.

[3] H. Coates and D. Edwards , The 2008 Graduate Pathways Survey (Australian Council for Educational Research [ACER], 2009).

[4] S. O'Shea , P. Lysaught , J. Roberts , and V. Harwood , “Shifting the Blame in Higher Education – Social Inclusion and Deficit Discourses,” Higher Education Research and Development 35, no. 2 (2015): 322–336, 10.1080/07294360.2015.1087388.

[5] R. Kent , G. Byrne , J. Stacy , and L. Charalambous , “Readers Panel: Should Nursing Students Be Paid for Clinical Placements Beyond COVID‐19?,” Nursing Standard 36, no. 2 (2021): 12.

[6] S. Smith , C. Smith , and M. Caddell , “Can Pay, Should Pay? Exploring Employer and Student Perceptions of Paid and Unpaid Placements,” Active Learning in Higher Education 16, no. 2 (2015): 149–164, 10.1177/1469787415574049.

[7] K. Hoskyn , C. Cameron , P. R. Lucas , et al., “Paid and Unpaid Work‐Integrated Learning: Challenges and Opportunities,” in The Routledge International Handbook of Work‐Integrated Learning, 3rd ed. K. E. Zegwaard and T. J. Pretti (Routledge, 2023), 548–562, 10.4324/9781003156420-39.

[8] Z. Wang and I. Crawford , “Who Are Gaining the Highly Paid Elite Placements in UK Higher Education?,” Studies in Higher Education 44, no. 11 (2019): 1960–1974.

[9] “Victorian Medical Radiations Practitioner's Education Trust,”.

[10] M. C. Shanahan and T. N. Akudjedu , “Australian Radiographers' and Radiation Therapists' Experiences During the COVID‐19 Pandemic,” Journal of Medical Radiation Sciences 68, no. 2 (2021): 111–120, 10.1002/jmrs.462. Epub 2021 Feb 15. PMID: 33590670; PMCID: PMC8013350. 33590670 PMC8013350

[11] K. Powers , “Faculty Perceptions of the Impact of the COVID‐19 Pandemic on New Graduate Nurses' Transition to Practice: A Qualitative Study,” Journal of Professional Nursing 43 (2022): 33–41, 10.1016/j.profnurs.2022.09.003. 36496242 PMC9484984

[12] A. Steward , S. Lacey , A. Gray , C. Parsons , K. Thompson , and N. Anderson , “The Ongoing Impact of COVID‐19 on the Clinical Education of Australian Medical Radiation Science Students,” Journal of Medical Radiation Sciences 72, no. 2 (2025): 193–201, 10.1002/jmrs.847. 39805227 PMC12159244

[13] Sheffield Hallam University , “Diagnostic radiographer degree apprenticeship,” https://www.shu.ac.uk/study‐here/higher‐and‐degree‐apprenticeships/health‐and‐social‐care/diagnostic‐radiographer.

[14] HCPC (Health and Care Professions Council) , “Degree apprenticeships in radiography: Guidance for practice‐based learning,” https://www.hcpc‐uk.org.

[15] T. Sevens , J. Nightingale , and N. Ali , “Degree apprenticeships for the radiography profession; are clinical departments ready?,” Radiography 28, no. 1 (2022): 75–79, 10.1016/j.radi.2021.08.001. [Correction added on [29 May 2026], after first online publication: Reference has been updated to the correct reference] 34456136

[16] D. Baker , “Potential implications of degree apprenticeships for healthcare education,” Higher Education, Skills and Work‐Based Learning 9, no. 1 (2019): 2–17. [Correction added on [29 May 2026], after first online publication: Reference has been updated to the correct reference]

[17] A. Fuller and L. Unwin , “Fostering Workplace Learning: Looking through the Lens of Apprenticeship,” European Educational Research Journal 2, no. 1 (2003). 10.2304/eerj.2003.2.1.9. [Correction added on [29 May 2026], after first online publication: Reference has been updated to the correct reference]

[18] “Department of Education Skills and Employment,” https://www.dewr.gov.au/skills‐education‐and‐employment/resources/see‐discussion‐paper‐2023.

[19] J. M. Longman , F. L. Barraclough , and L. S. Swain , “The Benefits and Challenges of a Rural Community‐Based Work‐Ready Placement Program for Allied Health Students,” Rural and Remote Health 20 (2020): 5706, 10.22605/RRH5706. 32611194

